# Moral distress among healthcare professionals in long-term care settings: a scoping review

**DOI:** 10.1186/s13010-025-00171-5

**Published:** 2025-06-12

**Authors:** Floor Vinckers, Elleke Landeweer

**Affiliations:** 1 University Medical Center Groningen, Groningen, Netherlands; 2 University of Humanistic Studies, Utrecht, Netherlands

**Keywords:** Moral distress, Healthcare professionals, Long-term care, Scoping review

## Abstract

**Aim:**

To explore the body of knowledge available regarding the moral distress of healthcare professionals in long-term care settings, focusing on influencing factors and strategies to cope with moral distress.

**Design:**

Scoping review.

**Methods:**

This scoping review follows the guidelines of the PRISMA-ScR protocol (Tricco et al. 2018) Searches were done using a strategy that included MeSH terms and free text terms.

**Data sources:**

Data sources were PubMed, CINAHL, Psychinfo and Embase. Searches were done in October 2023 without any date restrictions.

**Results:**

Eight articles were included in this review. Moral distress can impact the wellbeing of healthcare professionals. Influencing factors of moral distress of health care professionals appeared to be lack of resources, lack of communication and incongruence with colleagues. Strategies to cope with moral distress were talking about ethical issues with others, receiving support from colleagues and managers, and seeking support from outside the team or organization. Individual healthcare professionals relied on their personal characteristics or their professional identity and used rationalization, distancing themselves or acceptance of the situation to cope with their moral distress.

**Conclusion:**

Moral distress of healthcare professionals in long-term care settings appears not differently experienced than moral distress among healthcare professionals in other healthcare settings. This can be beneficial in learning from each other, but also raises the question whether moral distress is too broadly defined.

**Impact:**

This review addressed the scope and experiences of moral distress in long-term care settings. Future research can contribute to further insight into if and how specific features of long-term care are of influence on moral distress and formulate tailored strategies to lessen moral distress.

**Reporting method:**

PRISMA-ScR.

**Supplementary Information:**

The online version contains supplementary material available at 10.1186/s13010-025-00171-5.

## Introduction

Healthcare professionals are often confronted with morally complex situations that may cause conflicts in values and/or moral doubt. Conflicts and doubt can rise, for example, in situations when the organization’s policy chooses economics and efficiency over quality of care [17, 19, 33], when healthcare professionals feel undervalued in their contribution and expertise [19, 33], in case of poor communication within teams [7, 19, 33] or due to insufficient availability of (labor)resources [7, 19]. These complex situations can lead to moral distress among healthcare professionals.

Philosopher Andrew Jameton coined the concept of moral distress in 1984 as ‘the experience of knowing the right thing to do while being in a situation in which it is nearly impossible to do it’ [14, 16]. Since then, the concept of moral distress is well debated in scientific literature, discussing its scope, its relatedness to other concepts and its usefulness for the healthcare practice [20, 21]. Despite the fact that the scope of moral distress is not clearly defined and can be viewed as an ‘umbrella term’[12], there seems to be consensus it addresses a significant problem within healthcare. It is recognized that moral distress can have major impact on healthcare professionals. It can lead to healthcare professionals feeling frustrated, being negative about their job, sleeplessness and falling ill or burned-out [12, 15, 33]. In addition, moral distress is known to be a cause for healthcare professionals to ultimately leave their job, and therefore likely to add to continually growing staff shortages in healthcare worldwide [17, 34, 35].

Addressing moral distress in long-term care settings is urgent as the practice is confronted with increasing shortages of staff and cuts in finances [6, 11]. This could contribute to more morally complex situations for healthcare professionals.

Although various reviews were performed on moral distress in short-term care settings, such as in clinical care [13, 23], intensive care [3], critical care [2, 19], and oncology care [8], to our knowledge there are no reviews that addressed the context of moral distress among healthcare professionals in long-term care settings, focusing on the practice of providing daily care.

Long-term care constitutes of care for a long(er) period of time, focusing on a patient’s personal daily care needs. Patients can be living within a facility receiving 24-h care but can also receive home-based care. Patients receiving long-term care can be in need of daily care due to chronic (cognitive) conditions, functional limitations or a combination of those two [25]. Settings where long-term care is provided are among others elderly care, mental healthcare, intellectual disability care, and youth care. In long-term care the relation between the patient and their caregivers is characterized by its longitude and close proximity [6]. Therefore, how moral distress is defined and experienced in long-term care settings may differ from care practices that focus on (short-term) cure.

## The review

### Aim

To explore the body of knowledge available regarding the moral distress of healthcare professionals in long-term care settings, focusing on influencing factors and strategies to cope with moral distress.

We identified the following research questions:

1. How is moral distress in long-term care settings defined?
2. How does moral distress affect healthcare professionals in long-term care settings?
3. Which strategies and interventions are developed that support healthcare professionals in long-term care settings to handle moral distress?

## Method

### Design

This scoping review refers to the methodological framework developed by Arksey and O’Malley [1] and follows the guidelines of the PRISMA-ScR protocol [30].

### PICO

First the research questions were translated into a PICO scheme (population, intervention, comparison and outcome). Healthcare professionals in long-term care settings (defined as all employees within an organization that contribute to daily care practices, such as care workers, nurses and youth workers) were defined as Population. In this review we focused our search on the settings elderly care, mental healthcare, intellectual disability care and youth care as these are the settings known for their long-term care features. The Intervention formulated was moral distress. Comparison was not specified, and could be any or none. Outcome was described as how healthcare professionals were affected by moral distress and what strategies were experienced as supporting.

### Search strategy

The second step was identifying relevant studies. The search strategy and database selection were defined, using the PICO framework. The search string included MeSH terms (Morals, Nurses, Long-term care, Residential facilities, Mental health services, Psychiatric nursing, Intellectual disability, Child welfare, Child protective services, Nursing homes and Health services for the aged), and free text terms (related to moral and ethical (di)stress, dilemmas and challenges, (health)care workers, caregivers, nursing (staff), and youth or child protection workers and group or care homes, assisted living facilities, mental healthcare, intellectual disability care, youth or child care and elderly care), using AND and OR operators. This was first developed in PubMed, and later restructured for the other databases we used: Embase, CINAHL and Psychinfo. The specific search query for PubMed is presented in Additional file 1.

### Search outcome

The search was conducted in the databases PubMed, Embase, CINAHL and Psychinfo. All searches were done in October 2023 and without any date restrictions. In total, 3141 articles were found. Duplicates were removed, leaving 2245 articles. The titles and abstracts of the articles were imported into the Rayyan screening tool and reviewed by the authors based on the inclusion and exclusion criteria, using the blind setting. Discrepancies between the reviewer’s decisions were discussed until agreement was reached on in- or exclusion and 40 articles remained for the next phase of selection. Since no results for youth care and intellectual disability care came up in this search, an additional search for moral distress in youth care was done in Google Scholar, leading to 1 more article manually added. No articles were found that corresponded to the criteria in the field of intellectual disability care.

The 40 selected articles, together with the one manually added article, were closely read by both authors and screened on eligibility based on if they included results on one or more of the research topics. After discussing and reaching agreement on discrepancies, a total of 8 articles were selected to be reviewed.

**Fig. 1:**
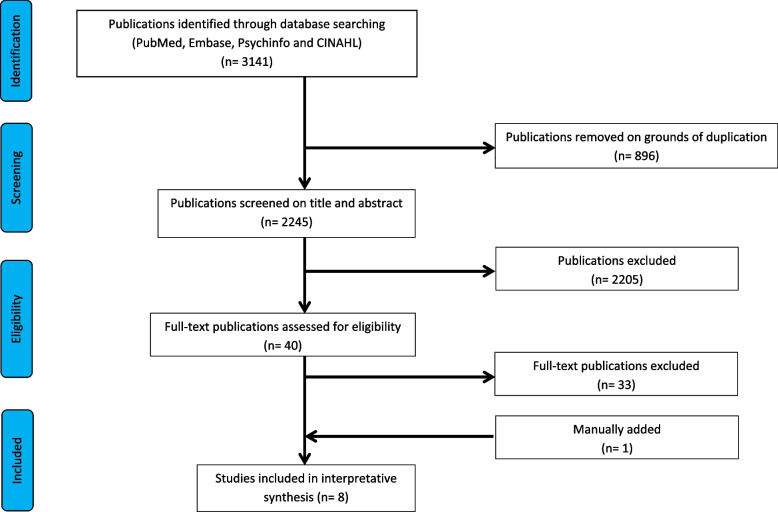
flow diagram of the study search and selection process

The study selection process is presented as a PRISMA flow diagram in Fig. 1.

### Inclusion and exclusion criteria

We included articles that met the following inclusion criteria:

- Published in English or Dutch
- Healthcare professionals as study population
- Situated in long-term care settings, including elderly care, mental healthcare, intellectual disability care and youth care
- Moral distress as subject of the research
- Based on empirical data

Exclusion criteria were settings outside of long-term care, including hospitals, and studies that were reviews, doctoral dissertations, editorials or conceptual papers.

### Quality appraisal

Quality appraisal of the articles was not performed as this scoping review was explorative.

### Data abstraction

Of the 8 remaining articles, fragments about authors, aim of the study, definition of moral distress, influencing factors of moral distress, effect of moral distress and helping strategies were selected and placed in a table. The abstracted information was discussed and adjusted by the authors in an iterative process.

### Synthesis

The information about each topic in the table (definition of moral distress, influencing factors of moral distress, effect of moral distress and helping strategies) was grouped and summarized by the first author using thematic analysis [5] and then aligned with the aims of the review, in dialogue with the second author.

## Results

Out of the 3141 identified articles, seven were eventually included and one was added manually, resulting in a total of eight included articles in this review. These articles ranged from publication in 2013 to 2023 and were conducted in different countries in Europe, America and the Middle-East, including Finland, Italy, United Kingdom, Canada and Iran. None of the included studies were conducted in the field of intellectual disability care. Most of the studies were conducted in either elderly care [9, 31, 36] or mental healthcare [22, 27, 28]. One of the articles focused on female healthcare professionals in general [26] and one article focused on the field of youth care/social welfare [18]. Most of the studies applied a qualitative research method, using semi-structured interviews. One study was quantitative, using survey data collected with an electronic questionnaire [18].

Most studies focused on factors that influenced moral distress, how moral distress affected healthcare professionals and how they responded to moral distress. Some of the studies also included strategies that according to healthcare professionals helped them to reduce moral distress [9, 22, 26, 31, 36], one study focused entirely on how healthcare professionals coped with moral distress [28]. An overview of outcomes based on these items is presented in Table 1.

**Table 1:** Overview of included articles

Authors, title (year) country	Aim of the study	Definition of moral distress	Influencing factors of moral distress	How moral distress affects healthcare professionals	Helping strategies
Edwards MP, McClement SE, Read LR. *Nurses'responses to initial moral distress in long-term care*. (2013) [9] Canada	To examine and describe the way registered nurses working in long-term care respond to initial moral distress, including the nurses'perceptions of resources or supports that helped or had the potential to help them and factors that hindered them in their response	Initial moral distress [15]	• When treatments or interventions requested by family or other members of the teams seemed contrary to resident wishes/best interest • Negative effects on resident care due to lack of resources • If residents'dignity is diminished by care provided to them • The context of the situation matters	• Feeling isolated due to incongruent values with other team members • When healthcare professionals do not feel supported by managers or other individuals in leadership positions, they report feeling isolated, frustrated and despaired	• Opportunities to discuss moral distress with other healthcare team members and managers • Support from and being taken seriously by colleagues or managers in moving concerns forward through meetings or conversations • Access to sources outside the team or organization, such as objective others, library resources and education
Musto L, Schreiber R, Rodney PP. *Risking vulnerability: Enacting moral agency in the is/ought gap in mental health care.* (2021) [22] Canada	To explore how healthcare professionals in acute care mental health settings navigate ethically challenging situations, enact moral agency, practice in congruence with ethical standards and mitigate moral distress	Jameton [14]	• The inability to respond with respect, dignity and compassion towards people struggling with mental health issues • Lack of resources, including time and physical space for patients	Not studied	• Support from colleagues or leaders and being taken seriously in enacting moral agency • A team should feel professionally and emotionally safe enough to share ethical concerns • Team discussion to discern the ethical obligations and find boundaries • Seeking support from outside the team • Rationalizing your actions • Distancing yourself from patients • Living with the situation • Holding fast to professional identity
Smith J, Korzuchowski A, Memmott C, Oveisi N, Tan HL, Morgan R. *Double distress: women healthcare providers and moral distress during COVID- 19*. (2023) [26] Canada	To identify types of moral distress among women healthcare professionals during COVID- 19 pandemic; to explore how feminist political economy might be integrated into the study of moral distress	Morley et al. [21]	• Female healthcare professionals can experience moral distress in response to both paid and unpaid care work • Staffing shortages, hindering the ability to provide quality care and dedicate time to the resident • Lack of communication or conflicting information	• Mental health issues • Burnout • Physical issues (heart attack)	• Going into counseling or therapy • Drawing on professional pride and fulfillment to resist moral residue • Awareness that female healthcare professionals can experience a double layering of moral distress in both unpaid care work and paid care work
Tavakol N, Molazem Z, Rakhshan M, Asemani O. *Moral Distress in Iranian Psychiatric Nurses: A Content Analysis*. (2022) [27] Iran	To investigate the causes of moral distress in Iranian psychiatric nurses	Jameton [14]	• Lack of professional competence • Organizational culture • Individual factors • Environmental and organizational factors: ○ high workload ○ lack of staffing ○ facility and space constraints • Weakness in professional and effective communication • Observation of moral dilemmas by nurses	Not studied	Not studied
Tavakol N, Molazem Z, Rakhshan M, Asemani O. *Strategies of Iranian Psychiatric Nurses to Deal with Moral Distress*. (2023) [28] Iran	To investigate the mechanisms and strategies of psychiatric nurses in dealing with situations of moral distress	Jameton [14]	• Working with underactive colleagues • Observing the mistakes of colleagues • Use of violence in dealing with aggressive patients • Weakness in establishing proper communication at the level of patients and colleagues	Moral distress causes distructive feelings such as remorse, guilt, physical problems, and lack of motivation	• Follow up and report to colleagues or head nurse • Take away the factors that cause moral distress by: ○ Strictness and punishing colleagues who do not perform their duties ○ compensating or correcting others'mistakes ○ modifying work assignment methods ○ strengthening anger management skills ○ committing to religious beliefs ○ improving communication with patients by listening and showing empathy • Strengthen professional communication with colleagues • Managerial support for nurses by valuing their opinions and their experience
Villa G, Pennestri F, Rosa D, Giannetta N, Sala R, Mordacci R, et al. *Moral Distress in Community and Hospital Settings for the Care of Elderly People. A Grounded Theory Qualitative Study*. (2021) [31] Italy	To analyze the experience of moral distress in all types of professionals providing daily care to elderly patients and residents, including both acute and long-term care facilities	Jameton [14]	• Lack of resources ○ scarcity of operators ○ the impossibility to dedicate time to the elderly • Difficult communication with relatives or caregivers	• Feelings during the morally distressful event: ○ helplessness ○ fear of consequences • Feelings fter the moral event: ○ satisfaction once the right action was performed ○ experiencing failure and overthinking • Physical consequences: hypertension and fatigue • Psychological consequences: feeling isolated or distracted	• Relying on group morality, sharing feelings and choices with colleagues and the team • Personal characteristics like soothing behavior, tranquility, optimism and faith • Having professional experience • Support and trust from leaders • Psychological support • Relational skills: being responsive • Organizational factors: hiring more operators and keeping each responsibility clear
Young A, Froggatt K, Brearley SG. *'Powerlessness'or'doing the right thing'—Moral distress among nursing home staff caring for residents at the end of life: An interpretive descriptive study*. (2017) [36] United Kingdom	To understand how NH staff experience moral distress when caring for residents during end of life (last 12 months)	Peter&Liaschenko (2013) [24]	• Poor communication with all concerned • Poor relationships • Incongruent values with others involved in care, leading to feeling powerless and unable to influence care decisions • Fear of the consequences of making a mistake or a wrong decision	Not studied	• Communicating with all concerned • Understanding the different values held by individuals • Working together and valuing the opinions and suggestions of those who are involved
Mänttäri-van der Kuip M*. Moral distress among social workers: The role of insufficient resources*. (2016) [18] Finland	To examine reactive moral distress among Finnish social workers employed in public social welfare services, both to illuminate this understudied phenomenon in the field of social welfare and to explore the role of perceived resource insufficiencies in explaining it	Reactive moral distress [15]	• Resource insufficiency: ○ increasing budget constraints ○ increasing work overload	• Reduced enthusiasm, inspiration, pride and vigor Compared to colleagues who did not experience moral distress: • less willing to continue in their post • more frequently on sick leave • less often have positive work-related experiences	Not studied

We start with describing how moral distress is defined in the studies, including the factors that are reported as influencing moral distress. After that, we present an overview of how moral distress affects healthcare professionals and lastly, we summarize which strategies are mentioned as beneficial in coping with moral distress.

### How moral distress is defined

All except two studies [26, 36] adopted the definition of moral distress by Jameton as starting point for their research. In 1984, he defined moral distress as the situation where a healthcare professional may know what is the right thing to do, but is constraint by institutional factors, making it impossible to act upon what is considered as the right course of action [14]. In 1993, Jameton extended his definition and distinguished between initial and reactive moral distress. Initial moral distress was described as the feelings of frustration, anger, and anxiety healthcare professionals can experience when faced with institutional obstacles and conflicts with others about their values. Reactive moral distress concerned the distress that people feel when they did not act upon initial moral distress [15]. One study focused on how healthcare professionals responded to initial moral distress [9]. One other study addressed reactive moral distress, described as: ‘a lingering state that impairs wellbeing over a longer term’ [18]. Of the two studies that did not adopt the definition of Jameton as starting point for their research, Young et al. [36] drew on the evolved definition of moral distress by Peter & Liaschenko [24], stating that moral distress is ‘a challenge that arises when one has an ethical or moral judgement about care that differs from that of others in charge’ [24, 36]. The second study defined moral distress according to the definition of Morley et al. [20] that describes moral distress as a combination of (1) the experience of a moral event, (2) the experience of psychological distress and (3) a direct causal relation between (1) and (2) [20, 26]. Although the scope of how moral distress is defined varies, all definitions of moral distress that are used centralize healthcare professionals’ inability to practice according to their moral standards.

### Influencing factors of moral distress

The studies show that healthcare professionals’ experiences of moral distress can be influenced by a variety of factors. Edwards et al. [9] found that often it is not just the topic of conflict itself that is the source of moral distress, but also its related contextual features, such as preexisting relationships with people involved, time available to respond, the healthcare professionals’ certainty what the right action would be as well as the readiness of people to talk to about the situation [9]. Smith et al. [26] found that female healthcare professionals experienced moral distress in response to both paid and unpaid care work, suggesting implicit gender norms as an additional source of moral distress [26]. Young et al. [36] reported that incongruent values with others can lead to healthcare professionals feeling powerless and unable to influence care decisions, which in turn causes moral distress. In addition, fear of the consequences of making a mistake or wrong decision was found as contributing to moral distress [36].

In most of the studies lack of resources was mentioned as factor of influence on causing moral distress [9, 18, 22, 26, 27, 31]. It mainly refers to shortage in staff, hindering the ability to provide quality care [9, 26] and dedicate time (attention) to the resident [26, 27, 31], causing moral distress. In some cases, lack of professional competence was mentioned as a risk factor for making mistakes, which creates moral distress [27]. One study mentioned the architecture and size of the building as lack of resources, with insufficient space to move around or recreate, resulting in overcrowded wards that caused moral distress among healthcare professionals [27]. One study focused solely on resource insufficiencies such as budget constraints and experienced work overload as a cause for moral distress [18].

Lack of communication was mentioned as another influencing factor for moral distress in multiple studies [26–28, 31, 36]. This included poor communication within the organization, which could lead to lacking information needed to provide the right care [26, 36], flaws in communication with relatives, for example not being on the same page regarding treatment [31, 36] and lack of proper communication towards patients, treating them with aggressiveness [27] or without empathy [28]. Poor communication between colleagues leading to arguments in the team was also mentioned as a factor causing moral distress [27, 28]. In addition, communication difficulties between hospital and nursing homes and GPs was reported as possible cause of moral distress [36].

How colleagues act can inflict moral distress as well, as stated in several studies. Examples are colleagues being underactive [28], showing lack of competence or discriminating patients [27], as well as making mistakes or acting aggressively or violently towards residents [27, 28]. Colleagues are also seen as playing a part in causing moral distress when having views on treatments or interventions that contradict with the resident’s wishes or his/her best interest, as advocated by other members in the team [9]. In addition, when the resident’s dignity is at risk was reported as a possible cause for moral distress among healthcare professionals [9, 22].

### How moral distress affects healthcare professionals

Five articles reported on how moral distress affected healthcare professionals in their study [9, 18, 26, 28, 31]. First, moral distress can affect healthcare professionals emotional wellbeing. Mänttäri- van der Kuip [18] reported about reduced enthusiasm, inspiration, pride and vigor after moral distress [18]. Tavakol et al. [28] mentioned moral distress causing remorse, guilt and lack of motivation [28]. Villa et al. [31] described both emotions of helplessness or fear of consequences during a morally distressful event and emotions that came up after experiencing a moral event, from feeling satisfied once the right action was taken to experiencing failure when this was not the case [31].

Second, studies reported that moral distress can have physical and psychological implications for healthcare professionals. Mentioned are frequent sick leave [18], hypertension, fatigue [31], heart conditions or burn out [26], as well as mental health issues [26], a decline in positive work-experiences [18], being distracted [31] or feeling isolated due to incongruent values with other team members [9, 31]. When healthcare professionals do not feel supported by managers or other individuals in leadership positions, they report feeling isolated, frustrated and despaired [9].

Third, one study reported that moral distress can also affect the care organization. Mänttäri- van der Kuip [18] found that of the healthcare professionals who experienced reactive moral distress, 42.4 per cent were not willing to continue in their current post [18].

### Strategies and interventions to handle moral distress

Several studies described that healthcare professionals developed coping strategies that helped them to mitigate moral distress. While Villa et al. [31] report that personal characteristics of healthcare professionals such as tranquility, optimism, and faith and having professional experience and relational skills, are of influence how moral distress is mitigated [31], various studies describe it is also helpful to talk about moral distress with other members of the team or with their manager [9, 28]. Together healthcare professionals may discern ethical obligations and professional boundaries, individually healthcare professionals can rationalize their actions, distance themselves from patients [22] decide to acquiesce in the situation for emotional and psychological self-protection [22] and/or draw on their professional identity, pride and fulfillment [22, 26]. Also support from outside the team or organization is mentioned as beneficial [9, 22] as well as going into counseling or getting psychological support [26, 31]. Tavakol et al. [28] found that nurses coped by trying to take away the factors that caused moral distress, for example by compensating mistakes of their colleagues or being strict and punishing them for not performing their duties, modifying work assignment methods, strengthening their own anger management skills, improving communication with patients or committing to religious beliefs [28].

Several conditions are mentioned that should be in place to handle moral distress. Support from management is one aspect that is considered as vital to improve the ethical climate within teams and with that to provide an environment in which healthcare professionals feel comfortable to address their moral concerns. Participants who were supported by colleagues or leaders and felt being taken seriously in enacting moral agency noted they were better able to handle moral distress [9, 22, 28, 31, 36]. Healthcare professionals need to feel being taken seriously and that their opinions matter [9, 22, 28, 36]. When a team feels professionally and emotionally safe to share ethical concerns, healthcare professionals are more willing to seek advice from their team members in understanding the situation and coming up with solutions on how to deal with it [22]. Working well together as a team could contribute to reducing moral distress [36] and improves by strengthening professional communication with colleagues [28]. To make shared moral decisions as a team can also prevent moral distress [31]. Yet, even if team discussions do not resolve the conflict to the nurses’ satisfaction, they were viewed as beneficial because they provided a way for nurses to have their concerns heard and recognized [9]. In addition, Young et al. [36] mention that moral distress could be reduced if healthcare professionals develop mutual understandings into why others hold different values and emphasizes the importance of an open communication on ethical issues [36]. In addition, in the study of Villa et al. [31] it is mentioned that the organization should provide a clear structure of responsibilities and that hiring more healthcare professionals could reduce moral distress [31].

## Discussion

With this review we tried to develop insight into how moral distress is defined in studies within long-term care settings, how it affects healthcare professionals and which strategies can help to cope with moral distress. While the definition of moral distress appears fluid, in general, all studies relate it to situations where healthcare professionals are hampered to act in accordance with their ethical standards. Factors that were found as contributing to moral distress were related to shortage of resources (availability of staff, work overload, lacking sufficient competences), poor communication within and between organizations as well as between colleagues, patients and families. These factors appear not specific to long-term care settings. In other healthcare practices, similar sources of moral distress were found. Shortage of nursing staff and (working with) insufficiently qualified nurses were also mentioned as sources of moral distress of nurses in surgical care [7], and in reviews within intensive care [3] and clinical care [13, 23]. The review of Arnold et al. [2] as well as of McAndrew et al. [19] on moral distress in critical care found that behavior of colleagues is also a factor causing moral distress for emergency and critical care nurses. Examples were colleagues crossing professional boundaries or witnessing malpractice of a colleague [2, 19]. McAndrew et al. [19] also found that communication problems between nurses, patients, families and physicians during end-of life decision-making were frequently described as a source of moral distress [19].

Our review distinguished various ways moral distress affected healthcare professionals, addressing emotional wellbeing of healthcare professionals (i.e., reduced enthusiasm, fear, insecurity and frustration), causing physical and psychological implications (i.e., frequent sick leave, burn out), as well as affecting the ambiance in teams and care organizations. In other studies, addressing short-term care settings, we found similar effects of moral distress on healthcare professionals. Feelings of frustration, anger and fatigue [2, 13, 23] and leaving the job [7, 13, 19] were mentioned as consequences of moral distress among nurses working in surgical, critical and clinical care.

Strategies to mitigate moral distress emphasized the importance of healthcare professionals to talk about their moral distress with colleagues, and receive counseling and support. In other healthcare settings, reviews stress the importance of addressing moral distress in organizations [13] and advise to support and empower nurses to express their ethical concerns [7] but also highlight more intervention studies are needed to investigate the beneficence of strategies how to handle moral distress [2, 19, 23].

### Strengths and limitations

Although there have been various reviews on moral distress in healthcare, this is the first review that specifically focused on empirical studies on moral distress in the long-term care context. As long-term care refers to a range of care practices, making it difficult to demarcate, we have specified our search area into concrete settings of long-term care to capture settings where healthcare professionals support individuals who depend on them for daily care. This demarcation can be perceived as a limitation of this study, as daily care is also provided in other (short-term) care settings.

While our search strategy received many hits, most studies did not meet the inclusion criteria. Also, we did not retrieve a balanced distribution of studies between the specific long-term care settings. A possible explanation could be that in youth care as well as intellectual disability care less studies are performed addressing moral distress among healthcare professionals. As this is a scoping review, the focus was on a broad rather than a depth overview of information, which could mean that this review does not give a complete overview of all available literature on this topic.

Another limitation in this study is that the research questions of this scoping review imply that moral distress is considered as a negative phenomenon. This was further underlined by the selected studies. Yet, some studies mention moral distress can also have positive connotation, illustrating healthcare professionals are morally attuned [10, 29]. To limit the scope, we did not include this aspect in our review.

## Conclusion

Based on this scoping review we can conclude that moral distress among healthcare professionals in long-term care settings is not differently defined or experienced than moral distress among healthcare professionals involved in other healthcare settings (i.e. short-term care settings). At one hand this is a promising outcome as this means healthcare settings can learn from each other, for example developed strategies to mitigate moral distress within the various sectors can be exchanged. One example is the need for moral space and empowerment of nurses to express their ethical concerns. To realize an ethical climate in teams and organizations in which healthcare professionals would feel safe enough to share ethical concerns, support of management is vital. At the other hand, the outcome also leads to critical questions regarding the concept of moral distress. Is the definition of moral distress not too broad if it does not reveal specific differences in outcomes between long-term care versus short-term care settings? Morality understood as a social practice, emphasizes how our moral understandings are socially constructed [4, 32]. This implies that moral distress could vary within different social and cultural settings. Further research could contribute to addressing if and how features of long-term care can be of influence in how moral distress is experienced and if tailored strategies are needed to lessen moral distress in long-term care.

## Supplementary Information

Supplementary Material 1.

## Data Availability

The datasets used during the current study are available from the corresponding author on reasonable request.
