# Supplementary material for: Moral distress among healthcare professionals in long-term care settings: a scoping review
Source: Philos Ethics Humanit Med. 2025 Jun 12;20:8. doi: 10.1186/s13010-025-00171-5 (PMC12160104; doi:10.1186/s13010-025-00171-5)
Supplement: Supplementary file 1 — Supplementary Material 1. [file 13010_2025_171_MOESM1_ESM.docx]

**Appendix 1 Search query PubMed**

("Morals"[Mesh] OR “moral stress*”[tiab] OR “moral distress*”[tiab] OR “ethical distress”[tiab] OR “ethical stress”[tiab] OR “ethical conflict*”[tiab] OR “moral injur*”[tiab] OR “moral pain*”[tiab] OR “moral conflict*”[tiab] OR “moral dilemma*”[tiab] OR “moral challeng*”[tiab] OR “moral uncertaint*”[tiab] OR “moral constraint*”[tiab] OR “ethical challeng*”[tiab])

AND

("Nurses"[Mesh] OR “care provider*”[tiab] OR “care worker*”[tiab] OR “caregiver*”[tiab] OR “Nursing”[tiab] OR “nurse*”[tiab] OR “healthcare worker*”[tiab] OR “nursing staff”[tiab] OR “nursing population”[tiab] OR "youth worker*"[tiab] OR "child protection worker*"[tiab])

AND

("Long-Term Care"[Mesh] OR “long-term care”[tiab] OR “longterm care”[tiab] OR “ltci*”[tiab] OR "Residential Facilities"[Mesh] OR “Resident*”[tiab] OR “group home*”[tiab] OR “assisted living facilit*”[tiab] OR “care home”[tiab] OR "Mental Health Services"[Mesh] OR "Psychiatric Nursing"[Mesh] OR “mental health care”[tiab] OR "Intellectual Disability"[Mesh] OR “intellectual disabilit*”[tiab] OR “disabled person*”[tiab] OR “disability care”[tiab] OR "Child Welfare"[Mesh] OR "Child Protective Services"[Mesh] OR “childcare”[tiab] OR “child care”[tiab] OR “Youth work”[tiab] OR “child welfare”[tiab] OR “youth care”[tiab] OR "Nursing Homes"[Mesh] OR "Health Services for the Aged"[Mesh] OR “homes for the aged”[tiab] OR “nursing home*”[tiab] OR “elderly care”[tiab] OR “eldercare”[tiab])
